# Vaping cessation support in england: current provision, confidence, and barriers identified in a cross-sectional survey of healthcare providers

**DOI:** 10.1093/ntr/ntag034

**Published:** 2026-02-14

**Authors:** Sarah E Jackson, Yo Seop Byun, Eve Taylor, Lion Shahab, Harry Tattan-Birch, Katherine East, Sharon Cox, Hazel Cheeseman, Masuma Pervin Mishu, David Hammond, Jamie Brown

**Affiliations:** Department of Behavioural Science and Health, University College London, London, UK; Department of Behavioural Science and Health, University College London, London, UK; Department of Behavioural Science and Health, University College London, London, UK; Department of Behavioural Science and Health, University College London, London, UK; Department of Behavioural Science and Health, University College London, London, UK; Department of Primary Care and Public Health, Brighton and Sussex Medical School, University of Brighton and University of Sussex, Brighton, UK; National Addiction Centre, Institute of Psychiatry, Psychology & Neuroscience, Kings College London, London, UK; Department of Behavioural Science and Health, University College London, London, UK; Behavioural Research UK, Edinburgh, UK; Action on Smoking and Health, London, UK; Department of Epidemiology and Public Health, University College London, UK; School of Public Health Sciences, University of Waterloo, Waterloo, ON, Canada; Department of Behavioural Science and Health, University College London, London, UK; Behavioural Research UK, Edinburgh, UK

**Keywords:** vaping cessation, e-cigarettes, nicotine dependence, stop smoking services, health service providers, barriers to care, intervention delivery, England

## Abstract

**Background:**

Vaping prevalence has increased substantially in England in recent years. However, little is known about the demand for vaping cessation support, or about providers’ preparedness and perceived barriers to offering it.

**Methods:**

We conducted a cross-sectional online survey with stop smoking service and healthcare providers in England (*n* = 96). The survey collected information on service demand, knowledge and beliefs about e-cigarettes, training, and confidence in providing vaping cessation support, types of interventions offered, and barriers to delivering support.

**Results:**

Of the providers surveyed, 39.6% reported seeing clients seeking vaping cessation support at least weekly, but only 17.7% reported seeing someone daily. By comparison, 93.7% reported seeing clients seeking smoking cessation support at least weekly, and 76.0% daily. Fewer than half (44.8%) of participants offered vaping cessation support; 59.4% had received training to do so. The proportion reporting high confidence in providing vaping cessation support was greater among those who had received training (70.2% vs. 40.5%). Behavioral support was the most common vaping cessation intervention offered (41.7%), followed by nicotine replacement therapy (22.9%), with few providing prescription medications (varenicline/cytisine/bupropion; 0%–2.1%). When vaping cessation support was available, participants commented it was often shorter and less intensive than smoking cessation support. Key barriers included lack of commissioning or funding, lack of training and limited evidence-based interventions. Concerns were also raised that quitting vaping may increase risk of relapsing to smoking.

**Conclusions:**

Four in 10 healthcare professionals involved in providing smoking cessation support in England report at least weekly demand for vaping cessation support, but current provision is patchy and constrained by structural and resource-related barriers.

**Implications:**

Collectively, our findings suggest vaping cessation is an emerging area of service provision with demand from clients but limited tailored support. Given constrained public health budgets and the current lack of evidence on the cost-effectiveness of vaping cessation interventions, policy makers will need to determine whether and how meeting this demand should be prioritized within tobacco control strategies.

## Introduction

Vaping has become an increasingly prevalent form of nicotine use in England.^1,2^ While e-cigarettes are often used as a tool to reduce or stop smoking,^3,4^ evidence suggests that people tend to use them for longer periods than traditional nicotine replacement therapy (NRT).^5^ They are also increasingly used by people who have never regularly smoked.^6^ Vaping is less harmful than smoking but not risk-free.^7^ While supporting smoking cessation remains the public health priority, some people who vape may want to quit due to concerns about nicotine dependence, health risks, or the financial cost of long-term use.^8,9^ However, quitting vaping can be difficult due to the addictive properties of nicotine and may carry the risk of relapse to smoking.^10^ These factors highlight there may be an emerging demand for cessation services specifically tailored to support people who want to stop vaping.

Most clinical guidance and service models for nicotine cessation have been developed in the context of smoking.^11^ In England, people who want to quit smoking can access support from specialist stop smoking services, tobacco dependence advisors working across community and inpatient settings, and non-specialist healthcare professionals such as general practitioners, pharmacists, and dentists who follow a range of guidance provided by the National Institute of Health and Care Excellence (NICE) and the National Centre for Smoking Cessation and Training (NCSCT). By contrast, there has been comparatively little focus on helping people to quit vaping and there are no official NICE guidelines on how to support people to quit vaping, although the NCSCT has developed resources for services.^12^ As a result, there is limited understanding about the demand for vaping cessation support in England, the types of interventions offered, and whether services adapt their approaches compared with smoking cessation. Further, because many people quit smoking through vaping, vaping cessation interventions must be carefully tailored and nuanced to reduce the risk of relapse to smoking. Providers’ capacity to meet these needs may be shaped by perceptions of the harms of e-cigarettes, confidence in providing support, and practical and systemic barriers such as time pressures, limited funding, priority of needs, insufficient training, and gaps in evidence-based treatment options.^13-15^ Evidence on the effectiveness of interventions for vaping cessation is still emerging, with low-certainty or inconclusive findings regarding the impact of behavioral or pharmacological treatments on quit rates.^10^ Nonetheless, existing studies suggest that there is demand for vaping cessation support.^16,17^

Little is known about how providers in England view their preparedness or what additional training and resources they consider necessary to deliver vaping cessation support. This study addresses these gaps by examining the knowledge, attitudes, practices, and perceived barriers surrounding this support among stop smoking service and healthcare providers in England. In doing so, it provides evidence to inform workforce training and policy in this evolving area of nicotine use and cessation support.

## Materials and methods

### Design and sample

Data were collected via a cross-sectional online survey using the REDCap online survey platform. The survey primarily aimed to understand service provision for tobacco products, with questions about service provision for stopping vaping included at the end. Eligible participants were healthcare professionals aged ≥18 years, based in England, and involved in providing smoking cessation support (eg, stop smoking service practitioners and advisors). Recruitment took place between 25 June and 7 August 2025 via emails targeting stop smoking services (distributed by members of the research team [S.J. and E.T.], the NCSCT, and other contacts in tobacco control) and advertisements on social media (LinkedIn). Due to the anonymous nature of the survey and the use of multiple dissemination routes, including snowballing of invitations, it was not possible to determine the total number of individuals invited to participate and calculate a response rate. Participants could optionally provide their contact details to enter a prize draw to win one of six £50 vouchers. Recruitment primarily targeted stop smoking services and professionals working within tobacco control networks. As such, the sample largely reflects healthcare professionals involved in delivering commissioned smoking cessation support in England, rather than all potential providers who may encounter individuals seeking help to stop vaping.

A total of 132 people responded to the survey, of whom 96 (72.7%) were eligible for inclusion, completed the survey, and formed the analytic sample (of those excluded, one was not based in England, and the remainder did not provide sufficient data to be included in the study, with most dropping out before completing the initial section assessing participant characteristics). All participants were screened for (i) implausibly fast response times and (ii) duplicate email addresses, to identify potential automated “bot” responses or manual participant deception.^18^ No evidence of such issues was found.

Ethical approval was obtained from the UCL Research Ethics Committee (ID 0780); all participants provided written informed consent before completing the survey.

### Measures

Data were collected using a structured questionnaire designed to capture service providers’ characteristics as well as their knowledge, attitudes, and practices related to supporting people who vape. The measures are described below and provided in Supplementary File S1.

#### Participant characteristics

Information was collected on participants’ professional role, primary workplace setting, and location, and whether their service focused on particular population groups or had eligibility criteria for tobacco cessation support. Participants also reported the length of time they had been providing smoking cessation support, the typical duration of support their service provided, and how often their service was approached by people seeking help to stop smoking cigarettes. Finally, participants were asked to provide their ethnic group.

#### Service demand for vaping cessation support

Participants were asked: “How often do people approach your service looking for support to stop vaping?” Response options were: daily, less than daily but at least weekly, less than weekly but at least monthly, less than monthly but at least once a year, less than once a year, or don’t know.

#### Perceived harm of e-cigarettes

To assess perceptions of relative harm, participants responded to the question: “How harmful do you think e-cigarettes are compared to regular cigarettes?” Response options were: not harmful at all, a lot less harmful, a little less harmful, about the same, a little more harmful, a lot more harmful, or don’t know. This measure is consistent with other national surveys.^19^

#### Beliefs about cancer risk

Beliefs about health consequences were measured with the question: “Based on what you know or believe, does using e-cigarettes cause cancer?” Response options were: no, yes, or don’t know. This question was adapted from a measure used in the International Tobacco Control Project Four Country survey.^20^

#### Training in vaping cessation support

Training experience was assessed with the question: “Have you received any training in providing cessation support to people who vape?” Response options were: no, yes, or don’t know. If participants responded “yes,” they were asked to provide further detail with the open-ended prompt: “Please describe the types of training you have received in providing cessation support to people who vape.”

#### Confidence in providing vaping cessation support

Confidence was assessed with the question: “How confident do you feel in supporting people to stop vaping?” Response options were: not at all confident, not very confident, somewhat confident, very confident, extremely confident, or don’t know.

#### Provision of vaping cessation support

Participants were asked: “Do you offer support to people who want to stop vaping?”. Response options were: no, yes, or don’t know. Those answering “yes” were given a checklist: “What types of support do you currently offer to people who want to stop vaping? Please select all that apply.” Response options included: behavioral support, NRT (eg, gums, patches, lozenges, or sprays), varenicline (Champix), cytisine, bupropion (Zyban), advice on nicotine pouches as a cessation aid, referral to other support, and other.

#### Comparison with smoking cessation support

Participants were also asked: “Does the support you offer to people who want to stop vaping differ from the support you offer to people who want to stop smoking cigarettes?”. Response options were: no, yes, or don’t know. Those who answered “yes” were prompted to explain with the open-ended item: “Please explain how the support you offer differs.”

#### Barriers to providing vaping cessation support

Participants were asked: “Which, if any, of the following do you think make it difficult for you or your service to provide support for cessation of vaping? Please select all that apply.” Response options were: lack of time, lack of knowledge, lack of confidence, lack of training, lack of demand, lack of evidence-based treatment, lack of motivation/low priority, risk of relapse to tobacco use/smoking, other, and none. Those who answered “other” were prompted to explain with the open-ended item: “If you choose Other, please specify.”

#### Training and resource needs

Participants were asked: “What specific training or resources do you think would be helpful to provide better support for people who want to stop vaping?” Responses were collected in an open-ended format.

#### Awareness of referral pathways

Awareness of specialist pathways was assessed with the question: “Are you aware of referral pathways for specialized cessation support for people who vape in your area?”. Response options were: no, yes, or don’t know. If “yes,” participants were asked to explain in detail through an open-ended follow-up.

### Analysis

All analyses were conducted using SPSS version 29. The analyses were not pre-registered and should be considered exploratory.

Participant characteristics were summarized using frequencies and percentages for categorical variables and medians and interquartile ranges for continuous variables. Responses related to vaping cessation service demand, beliefs about harms and health risks, training, confidence, provision of support, and differences between smoking and vaping cessation approaches were summarized using frequencies and proportions. We used chi-square tests to explore associations between frequency of encountering clients seeking support for vaping cessation (daily vs. less often) and provision of support (yes/no), and between receipt of training for vaping cessation (yes/no) and confidence in providing support (low/moderate/high). Open-ended responses regarding training received, types of support offered, differences in support, barriers, and resources were coded inductively using descriptive content analysis to identify recurring ideas, which were summarized narratively. Raw open-ended responses are provided in Supplementary File S2.

## Results

### Sample characteristics

We analyzed data from 96 service providers across all regions of England (Table 1). Participants were largely drawn from stop smoking services and related tobacco control networks, which represent the main providers of structured behavioral and pharmacological support for nicotine cessation in England. Most (88.5%; *n* = 85) were specialist stop smoking or tobacco dependence advisors; others included a general practitioner and professionals in management, coordination, clinical, or pharmacy roles. Over half (56.3%) worked in community settings, and 42.7% focused on specific groups, most commonly pregnant women, people with mental health conditions, ethnic minorities, young people, and those in deprived areas. Some also supported people with learning disabilities, autism, homelessness, or substance misuse. Most services (61.5%) had eligibility criteria for clients to access smoking cessation support, such as being current smokers (ie, not exclusively vaping), meeting minimum age requirements (typically 12 or 18 years), living, working, or being registered with a GP in the service area, or belonging to specific groups (eg, pregnant or postnatal women, inpatients, or people with mental health conditions). Participants had a median of 4 years’ experience providing smoking cessation support (range: <1 to 26 years). Most (76.0%) reported that clients approached the service at least daily for support for smoking cessation and the typical duration of support provided for smoking cessation was 9–12 weeks (67.7%).

**Table 1:** Sample characteristics.

**Characteristic**	**% (*n*)** ^**a**^
Professional role	
Stop smoking/tobacco dependency advisor	88.5 (85)
Service manager/lead	5.2 (5)
Coordinator/advisor in local authority public health team	2.1 (2)
Clinical staff	2.1 (2)
General practitioner	1.0 (1)
Pharmacy support staff	1.0 (1)
Primary workplace setting	
Community	56.3 (54)
Hospital—inpatient	20.8 (20)
Hospital—outpatient	4.2 (4)
Primary care	2.1 (2)
Remote (eg, online, app, telephone)	15.6 (15)
Other (eg, multiple settings)	3.1 (3)
Service focuses on a specific population	42.7 (41)
Service has eligibility criteria for cessation support	61.5 (59)
How often clients approach service for smoking cessation support	
Daily	76.0 (73)
Less than daily but at least weekly	17.7 (17)
Less than weekly but at least monthly	6.3 (6)
Typical duration of support provided	
4–8 weeks	6.3 (6)
9–12 weeks	67.7 (65)
>12 weeks	26.0 (25)
Region of England	
North East	9.4 (9)
North West	7.3 (7)
Yorkshire and the Humber	5.2 (5)
West Midlands	17.7 (17)
East Midlands	25.0 (24)
East of England	3.1 (3)
London	8.3 (8)
South East	11.5 (11)
South West	2.1 (2)
All of England	10.4 (10)
Ethnic group	
White	79.2 (76)
Asian	16.7 (16)
Black	4.2 (4)
How long provided smoking cessation support (years), median (IQR); min–max	4 (8); <1-26

^a^Unless otherwise specified. IQR, interquartile range; min, minimum; max, maximum.

### Perceived harm and health risks of e-cigarettes

Table 2 summarizes perceptions of the harms and health risks of e-cigarettes. Most participants perceived e-cigarettes to be a lot less harmful than cigarettes (82.3%), with 10.4% indicating they were a little less harmful, 1.0% about the same, and 2.1% more harmful. Regarding cancer risk, 5.2% believed that vaping causes cancer, 63.5% believed it does not, and 31.3% were unsure.

**Table 2:** Perceived harm and health risks of e-cigarettes.

**Measure**	**% (n)**
Perceived harm compared with cigarettes	
Not harmful at all	3.1 (3)
A lot less harmful	82.3 (79)
A little less harmful	10.4 (10)
About the same	1.0 (1)
A little more harmful	1.0 (1)
A lot more harmful	1.0 (1)
Don’t know	1.0 (1)
Believe that e-cigarettes cause cancer	
Yes	5.2 (5)
No	63.5 (61)
Don’t know	31.3 (30)

### Demand for and provision of vaping cessation support

Table 3 shows the demand for, and provision of, vaping cessation support. When asked how often clients approached their service for support to stop vaping, 17.7% reported daily, 39.6% at least weekly, and 74.0% at least monthly. A small proportion reported not knowing the frequency (4.2%).

**Table 3:** Demand for and provision of vaping cessation support.

**Measure**	**% (*n*)**
Frequency of clients seeking support for vaping cessation	
Daily	17.7 (17)
Less than daily but at least weekly	21.9 (21)
Less than weekly but at least monthly	34.4 (33)
Less than monthly but at least once a year	19.8 (19)
Less than once a year	2.1 (2)
Don’t know	4.2 (4)
Offer support for vaping cessation	
Yes	44.8 (43)
No	53.1 (51)
Don’t know	2.1 (2)
Types of support offered for vaping cessation^a^	
Behavioral support	41.7 (40)
Nicotine replacement therapy	22.9 (22)
Advice on nicotine pouches	6.3 (6)
Referral to other support	5.2 (5)
Cytisine	2.1 (2)
Varenicline	1.0 (1)
Bupropion	0 (0)
Other	5.2 (5)
None of these	55.2 (53)
Support differs from smoking cessation^b^	
Yes	55.8 (24)
No	44.2 (19)

^a^Participants were asked to select all that applied.

^b^Among those who reported providing support for vaping cessation; valid percentages shown.

Around half (53.1%) of participants reported offering support to clients who want to stop vaping, with uncertain higher provision of support among providers who encountered clients seeking support for vaping cessation every day versus less often than this (64.7% vs. 40.5%; χ^2^ = 5.45, *p* = .066). Behavioral support was the most commonly reported intervention, followed by NRT. Some reported offering advice on nicotine pouches, referral to other support (eg, local stop smoking services, pharmacies, online support), or other types of support (eg, brief advice but no ongoing support, signposting to local services). Few offered prescription medications (varenicline, cytisine, or bupropion).

Of those who reported offering support for vaping cessation, 55.8% said the support they offer differs from the support they provide for smoking cessation. In open-ended responses (Supplementary File S2), participants reported that, for vaping cessation, their service offers behavioral support only, without NRT or other pharmacological products. Support was also reported to be often shorter or more limited, with fewer sessions than smoking cessation programs. Some reported focusing on gradually reducing nicotine strength in e-cigarettes to help clients taper to zero nicotine.

### Training and confidence in providing vaping cessation support

Table 4 summarizes training and confidence in providing support for vaping cessation. Overall, 59.4% of participants reported having received specific training. In open-ended responses (Supplementary File S2), NCSCT courses—delivered both online and in-person—were frequently mentioned. Many also mentioned in-house or service-specific training, often including guidance on behavioral support and nicotine reduction strategies. A few reported training from vape manufacturers or suppliers, providing product knowledge, troubleshooting, and advice on tapering nicotine strength. Others mentioned webinars, leaflets, and websites.

**Table 4:** Training and confidence.

**Measure**	**% (*n*)**
Received training in vaping cessation	
Yes	59.4 (57)
No	38.5 (37)
Don’t know	2.1 (2)
Confidence in providing support for vaping cessation	
Not at all confident	2.1 (2)
Not very confident	9.4 (9)
Somewhat confident	29.2 (28)
Very confident	33.3 (32)
Extremely confident	24.0 (23)
Don’t know	2.1 (2)

Confidence in supporting clients to quit vaping varied, with 11.5% reporting no or low confidence (“not at all” or “not very” confident), 29.2% moderate confidence (“somewhat confident”), and 57.3% high confidence (“very” or “extremely” confident). Confidence was higher among those who reported having received training (1.8% low, 26.3% moderate, 70.2% high) than among those who had not received training (24.3% low, 32.4% moderate, 40.5% high; χ^2^ = 22.60, *p* = .012).

### Barriers to vaping cessation support

Table 5 summarizes barriers to providing vaping cessation support. Of the list of barriers presented to participants, the most frequently endorsed included risk of relapse to smoking, lack of training, and lack of evidence-based treatment. However, around a third (36.5%) of participants also reported other barriers in open-ended responses, with the vast majority (34.4% of all participants) citing a lack of commissioning or funding for vaping cessation (Supplementary File S2). They commented that services are generally funded and targeted only for tobacco users and vaping cessation is not a priority, restricting access for people who vape and limiting interventions to brief advice or minimal behavioral support. When asked about other support available in their area, some reported being aware of referral pathways for specialized vaping cessation support, such as social prescribing teams or specific local stop smoking services (Supplementary File S2). A further barrier, noted by some participants, was that current messaging frames vaping primarily as an effective stop-smoking aid, which creates challenges in offering vaping cessation services without stronger evidence to justify the need.

**Table 5:** Barriers to providing vaping cessation support.

**Barrier**	**% (*n*)**
Risk of relapse to smoking	19.8 (19)
Lack of training	19.8 (19)
Lack of evidence-based treatment	16.7 (16)
Lack of motivation/low priority	13.5 (13)
Lack of time	12.5 (12)
Lack of knowledge	11.5 (11)
Lack of demand	9.4 (9)
Lack of confidence	6.3 (6)
Other	36.5 (35)
None	16.7 (16)

When asked what training or resources would be helpful for improving vaping cessation support, participants had mixed views in open-ended responses (Supplementary File S2). Some thought existing training was sufficient, while others emphasized the need for up-to-date evidence and guidance, including vaping-specific training modules and examples of best practice. Practical resources, such as step-by-step guides, leaflets, and client-facing materials, were seen as helpful tools to support behavior change. Participants also highlighted the need for specialist stop vaping support, youth-focused approaches to support children and young people to quit, and adequate funding to enable effective service provision.

## Discussion

This study provides insights into the knowledge, attitudes, practices, and perceived barriers to providing vaping cessation support in England among a sample of healthcare professionals who primarily provide smoking cessation support. Overall, our findings highlight both the current demand for vaping cessation support and substantial variability in how services approach this issue.

A total of 56% of these healthcare professionals reported relatively infrequent demand for vaping cessation, with clients approaching them less than weekly about support. A substantial minority—around one fifth—reported frequent demand with clients requesting vaping cessation support every day—far fewer than the number reporting clients requesting smoking cessation support daily (76%). Only around half indicated that their service offered vaping cessation support, with providers encountering daily requests for vaping cessation support appearing more likely to offer such support. Behavioral support was most commonly provided, followed by NRT, whereas prescription medications such as varenicline or cytisine were rarely offered, which may reflect that the evidence of their effectiveness for vaping cessation is currently uncertain.^10,21,22^ Some participants noted that their service only offers behavioral support for vaping cessation, meaning they may have been unable to offer pharmacotherapy rather than choosing not to. When support was provided, it was often shorter and less intensive than that provided for smoking cessation.

Most participants perceived e-cigarettes to be less harmful than combustible cigarettes, consistent with public health evidence^7^ but unlike the general population who mainly perceive e-cigarettes to be equally/more harmful than smoking.^23,24^ This is promising because it suggests that healthcare professionals involved in providing smoking cessation support have more accurate perceptions than the wider population. Training in vaping cessation was moderately common, with around 60% of participants reporting receipt of some form of training. Confidence in providing support varied, but was higher among those who had received training, underlining the value of specific training in vaping cessation. Nonetheless, gaps remain in both the consistency and coverage of training, with some providers reporting little or no formal preparation to support people who vape. Lack of training was therefore identified as one of the most common barriers to providing vaping cessation support. Many providers also reported uncertainty about effective, evidence-based interventions for vaping cessation. These findings are consistent with previous studies (predominantly in the United States) that have found lack of knowledge and training and insufficient scientific evidence on effective approaches to vaping cessation to be common barriers to offering support.^13^

Besides training and evidence gaps, participants identified several structural and practical barriers. Lack of commissioning and funding were particularly salient, reflecting the fact that services are typically resourced for smoking cessation and may lack capacity to deliver comprehensive support for people who exclusively vape. This echoes themes identified in previous qualitative studies with stop smoking service providers in the United Kingdom.^14,15^ Some providers were unable to offer any support, while others could only provide brief advice or fewer sessions than those offered for smokers. Concern about relapse to smoking was also highlighted, indicating the need for evidence-based approaches that do not undermine ongoing smoking abstinence. However, this concern should be understood in the context that most vapers accessing support were likely current or former smokers; as the proportion of never-smokers seeking help to quit vaping increases, relapse-related concerns may become less salient outside the traditional smoking cessation setting.

These findings point to a potential tension: formally commissioning vaping cessation within stop-smoking services could signal to people who vape that they “should” quit, potentially prompting some to attempt cessation before they are ready and increasing the risk of relapse to smoking. This underscores the need to weigh any unintended impacts of promoting vaping cessation against the consequences of not supporting people to stop, particularly in balancing the higher harms of smoking against the lower—but still present—risks associated with vaping.^7^ The framing of e-cigarettes as a stop-smoking aid adds further complexity. E-cigarettes remain the most popular and effective smoking cessation aid in England,^4^ and if services are perceived as promoting vaping cessation, this may discourage smokers from using e-cigarettes to quit, for fear that vaping will be just another addiction to overcome.^25^

Collectively, our findings suggest that vaping cessation is an emerging area of service provision with demand from clients but limited tailored support. Given constrained public health budgets and the current lack of evidence on the cost-effectiveness of vaping cessation interventions, policy makers will need to determine whether and how meeting this demand should be prioritized within tobacco control strategies. The development of official NICE guidance and greater dissemination of formal (eg, NCSCT) training, complemented by practical resources such as step-by-step guides, client-facing materials, and examples of best practice, could improve provider confidence and capability. Regular updates may be needed to reflect the evolving evidence base.^10^ Clear guidance and referral pathways would help standardize care, particularly for high-risk groups such as young people, pregnant women, or those with comorbidities. Commissioning and funding could better support vaping cessation to ensure equitable access, albeit this should not be at the expense of budgets for tobacco cessation. Care must be taken to balance promoting vaping cessation with supporting people who smoke and use e-cigarettes to quit, so as not to undermine this effective smoking cessation strategy.

Interpretation of these findings should be considered in the context of the current service landscape in England. At present, there is no clearly defined or standardized infrastructure for vaping cessation support, and limited data on the extent to which different services provide or commission such support. Stop smoking services remain the primary source of structured behavioral and pharmacological support for nicotine cessation, and participants were largely drawn from these services and related tobacco control networks. As a result, the findings reflect the experiences and perceptions of healthcare professionals working within existing smoking cessation services, rather than providing nationally representative estimates of vaping cessation provision across all potential service settings. This lack of a clearly mapped service landscape further underscores the exploratory nature of the study and the need for future research to more systematically characterize vaping cessation provision in England.

A key limitation of the study was the convenience sampling approach, which may not fully represent all healthcare professionals involved in vaping cessation. The study did not specifically aim to recruit healthcare professionals engaged in providing vaping cessation support, but rather focused on those delivering smoking cessation support. Consequently, the sample may not reflect the views and experiences of individuals directly engaged in vaping cessation. However, this can also be viewed as a strength, as a targeted focus on vaping cessation might have overstated the level of natural demand. The relatively small sample size also limited the ability to conduct meaningful subgroup analyses. Nonetheless, our findings provide new insights into vaping cessation provision in England, highlighting substantial variability and the lack of consistent training, resources, and commissioning in this area. In recognizing vaping cessation as a distinct area, policy makers and commissioners can help ensure services have access to training, resources, and guidance needed to respond to the evolving needs of nicotine users.

## Supplementary Material

ntag034_Supplemental_Files

## Data Availability

Data are available from the corresponding author on request.
