# Supplementary material for: Vaping cessation support in england: current provision, confidence, and barriers identified in a cross-sectional survey of healthcare providers
Source: Nicotine Tob Res. 2026 Feb 14;28(11):1806–13. doi: 10.1093/ntr/ntag034 (PMC13687881; doi:10.1093/ntr/ntag034)
Supplement: Supplementary file 1 [file ntag034_supplemental_files.zip › Supplementary_File_1_ntag034.pdf]

SECTION 1: PARTICIPANT CHARACTERISTICS

Q1. What is your job role?

0. Stop smoking / tobacco dependency advisor
1. GP
2. Pharmacist
3. Dental practitioner
4. Other (please specify job title and sector)

Q2. In what setting is your primary workplace?

0. Community
1. Hospital (outpatient)
2. Hospital (inpatient)
3. Other

Q3. Does your service/practice focus on a specific population group or groups (for example, young people, people who are pregnant, people from ethnic minority groups)?

0. No
1. Yes

Q3-1. If you choose 1. yes, Please specify which group or groups you focus on.

[Open-ended response box]

Q4. Does your service/practice have specific eligibility criteria for clients to receive support for tobacco/smoking cessation?

0. No
1. Yes

Q4-1.If you choose 1. yes, please specify the eligibility criteria.

[Open-ended response box]

This survey focuses specifically on services within **England**. If your workplace or service provision is located outside of England, please answer based on your primary location in England.

Q5. Where in England is your service/practice located?

0. North East
1. North West
2. Yorkshire and the Humber
3. West Midlands
4. East Midlands
5. East of England
6. London
7. South East
8. South West
9. Other

Q6. Please enter the first part of the postcode where your service/practice is located.

[Open-ended response box]

Q7. To the nearest year, how long have you personally been providing smoking cessation support?

[Open-ended response box]

Q8. What is the typical duration of tobacco/smoking cessation support that you personally provide?

0. Less than 4 weeks
1. 4-8 weeks

2. 9-12 weeks

3. More than 12 weeks

4. Don't know

Q9. How often do people approach your service looking for support to stop smoking cigarettes?

0. Daily

1. Less than daily but at least weekly

2. Less than weekly but at least monthly

3. Less than monthly but at least once a year

4. Less than once a year

5. Don't know

Q10. Which group do you consider you belong to?

1. Arab

2. Asian/Asian British - Bangladeshi

3. Asian/Asian British - Chinese

4. Asian/Asian British - Indian

5. Asian/Asian British - Pakistani

6. Asian/Asian British - Any other Asian background

7. Black - African

8. Black - Caribbean

9. Black - Any other Black / African / Caribbean background

10. Mixed - White and Asian

11. Mixed - White and Black African

12. Mixed - White and Black Caribbean

13. Mixed - Any other Mixed / multiple ethnic background

14. White - English / Welsh / Scottish / Northern Irish / British

15. White - Gypsy or Irish Traveller

16. White - Irish

17. White - Any other White background

18. Any other ethnic group

SECTION 4: AWARENESS AND SUPPORT PROVISION (VAPING)

Thank you for answering our questions about supporting people to stop using tobacco. We're also interested in whether you are supporting people to stop vaping electronic cigarettes (e-cigarettes).

Q86. How often do people approach your service looking for support to stop vaping?

0. Daily
1. Less than daily but at least weekly
2. Less than weekly but at least monthly
3. Less than monthly but at least once a year
4. Less than once a year
5. Don't know

Q87. How harmful do you think e-cigarettes are compared to regular cigarettes?

0. Not harmful at all
1. A lot less harmful
2. A little less harmful
3. About the same
4. A little more harmful
5. A lot more harmful
6. Don't know

Q88. Based on what you know or believe, does using e-cigarettes cause cancer?

0. No
1. Yes
2. Don't know

Q89. Have you received any training in providing cessation support to people who vape?

0. No
1. Yes
2. Don't know

Q89-1. If yes, please describe the types of training you have received in providing cessation support to people who vape.

[Open-ended response box]

Q90. How confident do you feel in supporting people to stop vaping?

0. Not at all confident
1. Not very confident
2. Somewhat confident
3. Very confident
4. Extremely confident
5. Don't know

Q91. Do you offer support to people who want to stop vaping?

0. No
1. Yes
2. Don't know

Q91-1. What types of support do you currently offer to people who want to stop vaping? Please select all that apply.

0. Behavioural support
1. Nicotine replacement therapy (e.g., gums, patches, lozenges, or sprays)
2. Varenicline (Champix)

3. Cytisine

4. Bupropion (Zyban)

5. Advice on nicotine pouches as a cessation aid

6. Referral to other support

7. Other

Q92. Does the support you offer to people who want to stop vaping differ from the support you offer to people who want to stop smoking cigarettes?

0. No

1. Yes

2. Don't know

Q92-1. If yes, please explain how the support you offer differs.

[Open-ended response box]

SECTION 5: BARRIERS AND FACILITATIORS FOR PROVIDING EFFECTIVE SUPPORT (VAPING)

Q93. Which, if any, of the following do you think make it difficult for you or your service to provide support for cessation of Vaping? Please select all that apply.

0. Lack of time

1.Lack of knowledge

2. Lack of confidence

3. Lack of training

4. Lack of demand

5. Lack of evidence-based treatment

6. Lack of motivation/low priority

7. Language barriers

8. Accessibility barriers

9. Risk of relapse to tobacco use/smoking

10. Other

11. None

Q94. What specific training or resources do you think would be helpful to provide better support for people who want to stop vaping?

[Open-ended response box]

Q95. Are you aware of referral pathways for specialised cessation support for people who vape in your area?

0. No

1. Yes

2. Don't know

Q95-1. If yes, please explain in detail

[Open-ended response box]
