# Supplementary material for: Vaping cessation support in england: current provision, confidence, and barriers identified in a cross-sectional survey of healthcare providers
Source: Nicotine Tob Res. 2026 Feb 14;28(11):1806–13. doi: 10.1093/ntr/ntag034 (PMC13687881; doi:10.1093/ntr/ntag034)
Supplement: Supplementary file 1 [file ntag034_supplemental_files.zip › Supplementary_File_2_ntag034.pdf]

Training

Have you received any training in providing cessation support to people who vape? If yes, please describe the types of training you have received in providing cessation support to people who vape.
**Comment**
at the start of using vapes with in the service the manufacturer of the brand we were going to use came in to explain - also had a person that came in to visit from a shop he was very knowledgeable
Behavioural and Prescribing NRT
Direct training and also attended online webinars regarding vaping.
From suppliers. Hints and tips to cutting down, reducing strength etc
Had training and researched into vaping and it's effects, mainly aimed at children. Don't feel there is any long term evidence to show the effects of vaping and if is will be damaging. The current research shows it to not be as damaging as smoking due to the lesser chemical in it, but there is conflicting evidence
Had training but our pharmacy have said no to us providing them
have attended the NCSCT training and training from Improving performance in Practice team.
How vapes can help them quit smoking, facts about vapes.
I have had in house training on E cigarettes.
I have leaflets to provide and information on the ASH Website to support that although not 100% safe Vaping is definitely less harmful than smoking tobacco.
I have looked at the online training by NCSCT but not in depth as I am not allowed to offer cessation to people who only vape. But if they smoke as well, I can offer them support.
In house training, NCSCT,
Information and training from vape company to help support transition from smoking to vaping
information on use, troubleshooting , impact
It was more a handout than specific training.
Just on the NCSCT
loads of training face to face, plus online stuff with NCSCT
Local training bespoke to my local stop smoking service
ncsct
NCSCT
NCSCT
NCSCT
NCSCT
NCSCT
NCSCT
NCSCT
NCSCT
NCSCT Swop2stop E-learning
ncsct e-learning
NCSCT in person
NCSCT previous manager sessions. NB people who vape are not eligible for our service as they are non smokers. we help smokers to quit with eCigs /vapes . some make the lifestyle choice to stay on them rather than titrate down liquid strengths and off

Supplementary File 2: responses to open-ended questions

NCSCT training
NCSCT training   Swap to stop training E learning
NCSCT training CBQ Method Behavioural Support
NCSCT training Stop smoking advisor training
NCSCT Training on Swap to stop
NCSCT training, Training from NENC, Boost learning
ncsct training. Provider training
NCSCT vaping mudule
NCSCT, in house, vape companies
NCSCT, NICE Guidance, in -house training
ncsct. in house training
NCST specialist module on vaping use. On Q88 , this is a difficult question to answer as there is no conclusive evidence (yet) on whether vapes can cause cancer. The picture may be very different in 20 years time.
ON LINE
online
Online training and demonstrations provided by our ECigarette company.
Online training Ncsct as well as in house training
our service has done the swap to stop so we are taught how to reduce after smoking but not to stop vaping
part of my 2 day Practitioners TDiP training
Reducing nicotine strength (titration and weaning), reducing time between vaping, not vaping at certain times of day to increase the time between vaping, staying occupied and busy, keeping hands busy with knitting puzzles, etc. and understanding nicotine dependence.
service specific training, NCSCT training and training fro. vape suppliers for specific product knowledge
Swap to Stop, NCSCT
Training from a vape company and also in house to encourage the reduction of strength in nicotine strength to a 0% and then behaviour change.
Training through ncsct, local vaping team
Via NCSCT and MI training
We have training for advisors to give advice on reducing vape usage
Webinars/Meetings NCST training/e-learning Courses

How support differs

Does the support you offer to people who want to stop vaping differ from the support you offer to people who want to stop smoking cigarettes? If yes, please explain how the support you offer differs.
**Comment**
Behaviour support only no nrt
Behavioural only
Behavioural support only to help quit vaping no products.
chat only. no nrt. reduction advice
don't provide NRT
For adults- signposting to NHS services and a one-off session. For children and young people, the service is the same for smoking and vaping
If a person was wanting to stop smoking you give them as much nicotine as they need/want but if they are wanting to stop vaping you want them to be reducing there nicotine so reduce the strength in the vape until they are down to a 0% and suggest that they leave it away from them so the temptation is not to use it at any given moment.
It will be unique to them. Eg. Youth vaping or wanting to be eventually nicotine free.
no NRT products
No NRT. Less sessions of support although this would be based on users confidence.
no products
not licenced to provide nrt to vapers
one off support
only a few sessions allowed focused on behaviour changes
There is no onward referral on hospital discharge.
we cannot provide nrt for people wanting to quit vaping- they would need to buy this themselves- we just offer the behavioural support
We do not provide NRT and we ALWAYS ensure quitting vaping will not result in the client reverting back to smoking
We do not support people to quit vaping on our smoking cessation programme, however we can offer them up to 3 appointments based in motivational interviewing to aid behaviour change.
We don't offer them vapes
We don't provide NRT or medication just behavioural support
We only offer NRT and behavioural support
We only provide behavioural support and up to 3 sessions in a 12 month period. As we consider vapes as a 'Cigarette cessation tool', I believe the stance we have from Public Health w.sussex is to support the use of vapes and those clients can't join our traditional, up to 12 week programme.
we use Nicotine analogues to cut vaping addiction
when someone is solely interested in using vape as a stop smoking aid, more information on the positive benefits and why its considered as better alternative is discussed. Also, discussion regarding the MHRA standards, safety, side effects, usage, short and long term effects, evidence based research is conducted.

Barriers

**Which, if any, of the following do you think make it difficult for you or your service to provide support for cessation of vaping? Please select all that apply. If you choose 10. Other, please specify:**
**Comment**
1. Vaping is considered as an effective first-line stop smoking aid. People willing to stop vaping are usually the ones who have been vaping for long and would like to come off or who have been advised by their respiratory consultants. 2. I also support youngsters to quit vaping if they have no smoking history and have been advised by their consultants to do so. They may suffer from childhood asthma or are simply nicotine addicted. 3. At times pregnant vapers are keen to quit because they are concerned about their baby's health - constant nicotine supply to the foetus.
a persons motivation
Again, it's not a apart of the funded programme on offer
As this is not funded as part of smoking cessation programme, we can only offer 3 appointments when clients may benefit from longer intervention and/or NRT with behavioural support
Commissioners do not allow us, our criteria is to support those using tobacco
Commissioning
Commissioning - most services are not commissioned to help with vaping cessation.
Funding
it isn't part of the commissioned service
Lack of funding. We only provide Nrt to those that smoke. However if a user downloads the app and is a vaper they can use the app features and we can provide hints and tips to help them reduce/stop vaping
may be funding.
money/funding, we arent given a budget for this
My service provides e-ciggs/vapes as a stop smoking aid/tool under the government's 'swap-to-stop' scheme allocation.
No funding for this service
not a target. not commissioned not a priority
Not commissioned
not commissioned to offer support
Not eligible for our service
Now being contracted to provide support.to this population
Our funding provider stipulates we provide for combusted tobacco only.
Our service is for tobacco dependency only, we can offer advice.
Our service is not commissioned to provide help to stop vaping
Providing support for cessation of vaping is outside our Service remit.
Restrictions on support and NRT supply by LA financing support for smokers
Service capacity only allows us to focus our delivery on those currently using tobacco products. Whilst our smoking prevalence is lowering we still have cohorts where our prevalence is high and we need to focus our efforts here. If we had further funding we would offer support to stop vaping but as yet due to LASSSFG we need to focus our delivery here
Stop Smoking Service is not commissioned to provide the support fpr ecig users.

The service is new and still getting going with the focus on smoking tobacco 1st - we do offer behaviour support to stop vaping with our Social Prescribers within the same team at the moment
The smoke free generation funding does not focus on clients who want to stop vaping (except young people) and is only targeted at tobacco users. Despite many people requesting help to stop vaping due to the health and financial impacts and their highly addictive nature we are unable to help them due to funding constraints.
Unable to provide/prescribe Vapes as a form of NRT as our Pharmacy will not approve this.
vaping seen as safer than smoking- focus on tobacco smokers first
we are not permitted to do so . they are deemed ineligible to take up the service.
we are told that our service does not provide support to people who vape, only tobacco smokers, in fact we offer e cigarette as an option for nicotine replacement as an aid to smoking cessation.
we only give support to tobacco , only give advise have they can stop vaping .
We provide e-cigs as an aid to stop smoking.
we regard vaping as a quitting smoking

What would be helpful

What specific training or resources do you think would be helpful to provide better support for people who want to stop vaping?
**Comment**
A acreditat regular update training
a clear protocol
a little A6/A7 size guide to hand out
A short step by step guide with tips
a specific service
A targeted specialist stop vaping advisor and groups specifically for vapers.
A training module and offering of the service.
Additional modules on NCSCT training
Advice on products to use as replacement for the nicotine and the habit
allow us to accept vaping clients that want to stop. also stop offering e cigarettes as a stop smoking aid as part of the programme.
Any
Any relevant training is fine. A research on the long term effects of vaping will help
awareness
Behavioural Support, such as CBT. CBQ method is helpful because it foes not use replacement nicotine to help people quit nicotine addiction.
Being able to give vapes
Being able to offer weekly behavioural support
Case studies from experienced vapers who have successfully quit
designated service and funding
don't know
easy access
effective treatment plans to quit vaping rather than one off advice in reducing nicotine content
Evidence based practise vaping isn't as harmful as smoking.   Any long term health effects it has had
Extra funding - they are non smokers so not entitled to full access on the app
F2F and teams
funding provision
general info on use and see vaping a stop smoking programme for a 12 week period so you support to cut down and come off vapes like you do cigs.
giving them NRT and behavioural support
Good to have more info on different vapes and nicotine levels
Groups to help people come out of the habit of vaping, changing habits
Having knowledge of vaping and how difficult it can be to stop using a vape. Knowledge of how to stop using a vape and training where possible.
Having specific stop vaping services targeting young people
Help to understand why people may wish to stop vaping as although it is less harmful some people wish to not use any nicotine or take anything into their bodies.
how to taper down and also get rid of hand to mouth habit

I think given the stance on vaping as a tobacco cessation tool being so positive, it's contradictory to offer something as a support tool for one service, and then offer a whole other service to the contrary. We would need to see more backing on the potential harmful effects of vaping in an evidence based format, which will possibly come from long term observational studies, however the technology is relatively new, and that isn't available yet.
If no nicotine then NRT no appropriate so what is
information on the various types of vapes available,   appropriate treatment for people who use refillable vapes, what is the equivalent NRT treatment for someone who vapes 100mg of e-liquid a week for example.
Interactive training sessions, ongoing support, sharing case studies, resources to share with clients, peer support sessions to discuss difficult cases and share best practice.
It is difficult because currently vaping is considered as an effective first-line stop smoking aid. We do not have enough research to conclude the harmful effects of vaping on lung health or in general.
leaflets, ap
long term side effects are not known so more information to be able to give advice would help
Mandatory VBA training for all Maternity team, not just Stop Smoking Practitioners.
maybe training on which method to use to help clients quit
More advice on how they work and eliquid strengths
More awareness for all on vaping and facts and how it is beneficial to help someone quit smoking and can help as long as it is a legalised vape and best method of practice on how to use a vape.
more clear in depth information and how it works.
More direct training and webinars and guides.
More education on nicotine dependence and how nicotine affects behaviour disturbance.
More evidence based practice/studies More in person training courses
More evidence based treatment facts and training
more information & training resources
More information about where to go for the service.
More information of what is in a vape and visual of how it works
more training and support
More training around quit aids
n/a
N/A
N/A
NCSCT
NCSCT training and detailed guidelines on monitoring use
NCSCT Training platform
never had any training on supporting people to stop vaping   Until vapes are promoted as dangerous, there will be no training. In fact, we are told that vapes are less harmful than smoking but how do you know? I have had patients who really struggle breathing when vaping yet there is no promotion to advertise dangers of vaping. I personally hate vapes, the vapour is everywhere and really gets on my chest when I am out in public yet it seems to be socially acceptable?????
Nicotine Replacement Therapy and more training on quitting vaping and more information on the dangers of vaping.
Non. Feel like there is enough training available
none
None

nothing, I think there is a lot out there
NRT for the early stages of reducing vaping
online portal
online resources
People need an understanding of the strengths of vape they are buying and understand how to lower themselves down to wean off the nicotine
Prescribing relevant medications
QR code reader fact sheet
Quick and to the point videos to watch with ideas and suggestions on how to do it with minimal withdrawals
Relapse support and to offer support to women / partners postnatally who think it is ok to start smoking again once baby is born. I guess more Passive smoking training / approach would be beneficial.
Resources to send to clients with recommendation and guidance to help them quit vaping. We currently provide our own.
Services are not funded to help people quit vaping. The focus is, and quite rightly should be on helping people stop smoking.
Some brief training around best ways to do this
special training on Vaping by NCSCT
Specific standard programme
standard treatments should suffice . it would be good to have champix back in the tool kit!
Stop Smoking Services need to be commissioned to provide the service by ICB
there are lots of misconception that circulates around vaping. More positive stories about people quitting smoking through vaping would be beneficial. Training for staff is also essential if they need to communicate it with their patients.
There is great advice already on NHS better Health website which we use to give VBA and refer clients to it.
There is very limited training in NCSCT. Better to have detailed one.
there needs to be more research done on the harms of vaping. Also more in depth training about how to quit and how this differs from cigarettes
To provide better support for people who want to stop vaping, the following training and resources would be especially helpful: Training Needs: Understanding nicotine addiction in vapers, including how it differs from smoking. Behavioural support techniques specifically tailored for people trying to quit vaping (especially young users). Training on withdrawal symptoms and relapse prevention for e-cigarette users. Up-to-date evidence on the safety, risks, and long-term effects of vaping. Engaging young people and adolescents, as this group is increasingly affected.
Training specific to helping people to stop vaping. Stop Smoking Services offering support to help people to stop vaping.
Vape cessation training Also it appears like we are not encouraged to help people stop vaping as vapes are so strongly encouraged by the NCSCT and others. It's like you cant be seen to actually want someone to stop vaping if you work in tobacco dependence treatment services.
Vaping cessation training however we are tobacco treatment advisors
Vaping harmful effects
We have covered this within our service and the team regularly receives MI training and behaviour changes training
We have regular training on this.
we need a plan to help people to stop vaping

We only provide support to people who smoke a tobacco products (12 week programme, extended by couple of weeks for clients with MH or extended by 4 weeks or a maximum of 12 weeks in total for SME clients. We support pregnant clients with NRT/ behavioural support until birth/ until 3 months postnatal if on regional incentive scheme for pregnancy. We only provide advice on stopping vaping - reducing vape liquid strength, allow longer breaks between vaping, putting vape out of reach, setting rules, distraction strategies. Maybe an online resource would be helpful, so that we can direct/ signpost clients / forward a link.
what product might help.
Workshops/slides/leaflets etc

Referral pathways

Are you aware of referral pathways for specialised cessation support for people who vape in your area? If yes, please explain in detail.
**Comment**
Another section of the Service that I work for ie Social Prescribing (separately funded), can help with behaviour change for vapers, but generally they don't feel confident with this and they can't offer Nicotine Replacement Therapy. Also they don't have training for most of the products you mention but they do have access to the NCSCT training on helping people quit vaping and they have all had health coaching training and training in behaviour change support (as do the Stop Smoking Service Staff).
community
I have an email and information leaflets that I can send to offer to support.
local community stop smoking service
Local services - some provide help depending on area
Local stop smoking services.
London SSS
NHS free app and tips how to stop vaping
One you lincolnshire
ourselves if pregnant and live well Wolverhampton for any one else
Self guided pathway and materials are provided, as we cant offer NRT, we advise only
Specialist referral, online referral, self referral- online, on the phone, text message, email or QR code
Stop smoking service
Via our Social Prescriber's (same team as us) they offer behavioural support but re reading the question it isn't specialised
we can refer patients to community where they have the option to opt for free vapes and behavioural support.
We provide this service in Barking and Dagenham and Havering
we would be the appropriate service for support
